# Effectiveness of post-acute care for patients with cerebral vascular disease in Taiwan between 2014 and 2023: a narrative synthesis

**DOI:** 10.3389/fresc.2025.1667253

**Published:** 2025-12-16

**Authors:** Cheng-Che Wu, Chang-Cheng Wu, Kuan-Chia Lin

**Affiliations:** 1 Department of Physical Medicine and Rehabilitation, Taoyuan General Hospital, Ministry of Health and Welfare, Taoyuan, Taiwan; 2 Institute of Public Health, School of Medicine, National Yang Ming Chiao Tung University, Taipei, Taiwan; 3 Institute of Hospital and Health Care Administration, School of Medicine, National Yang Ming Chiao Tung University, Taipei, Taiwan; 4 Community Medicine Research Center, National Yang Ming Chiao Tung University, Taipei, Taiwan; 5 Cheng Hsin General Hospital, Taipei, Taiwan

**Keywords:** post-acute care, effectiveness, functional outcome, quality of health care, stroke

## Abstract

**Objectives:**

To explore the overall effectiveness of the Post-Acute Care-Cerebrovascular Diseases (PAC-CVD) program in Taiwan, which was implemented in 2014.

**Data sources:**

A systematic search of databases, namely PubMed and Google Scholar, was conducted. Eligible studies published between Jan 2014 and June 2023 were included.

**Study selection:**

Studies included those that explored stroke care, involved post-acute care, were conducted in Taiwan, focused on an inpatient model of the PAC-CVD program, and had either a quantitative or qualitative design. In total, 23 articles were identified and included for narrative synthesis after complete examination.

**Data extraction:**

Multiple observers independently extracted the research articles, with their objectives focused on topics such as patient outcomes, quality of care, the influence of referral systems, cost-effectiveness, or outcome prediction for the PAC-CVD program.

**Data synthesis:**

The PAC groups showed significantly better performance in most functional outcome, quality of care, and cost-effectiveness indicators than the non-PAC groups. Patients with intra-hospital referrals or in partner hospitals had better outcomes. Younger age, ischemic stroke, and better baseline condition, especially in balance function, were strong predictive factors for stroke prognosis in the PAC program.

**Conclusions:**

The PAC-CVD program, implemented in Taiwan through the establishment of an integrated healthcare system and a change in payment systems, not only enhanced functional recovery and quality of life of acute stroke patients but also improved the quality of health care. The program also offered a more efficient and effective care model for acute stroke patients by reducing medical expenditures. However, the PAC program has also increased the workload of clinical healthcare professionals. The successful PAC-CVD implementation indicates the possibility of a standard rehabilitative care model for acute stroke patients, with expansion to other diseases or conditions possible after adjustments to the payment structure and workload.

## Introduction

Stroke is one of the most life-threatening neurological diseases, with continuously increasing prevalence worldwide. Approximately 17 million people suffer from strokes each year worldwide. It is also the second-leading cause of death and disability-adjusted life year globally (1, 2). Stroke causes different types, sizes, and locations of brain lesions, leading to various degrees and dimensions of impacts, including physical activity, extremities motion, sensation, speech, swallowing, cognition, and quality of life (QoL). It also results in disability and psychological sequelae, including depression, self-image, and social role disorders (3). The increasing number of survivors after strokes with disability is due to the increased prevalence and declined mortality of strokes (4). Stroke survivors often experience prolonged hospital stays, readmissions, and the need for long-term post-stroke rehabilitation after discharge. Long-term rehabilitation training required for functional recovery after a stroke (5), as well as residual disabilities, place a significant burden on families, communities, and the healthcare system.

Adequate rehabilitation training and care following acute medical treatment in stroke units play crucial roles in restoring physical functionality and improving overall patient QoL. Post-acute care (PAC) has been shown to reduce physical impairments and disabilities, improve QoL, facilitate functional recovery, and enhance the overall outcomes of patients (6). Additionally, it can reduce length of hospital stay (LOS) and occupancy of beds in large-scale hospitals, while also decreasing complications and readmissions. Furthermore, the introduction of PAC results in improved efficiency of medical resource allocation, lower healthcare expenditures (7), and less stress on healthcare systems. In Taiwan, acute stroke patients often experience prolonged hospitalization or readmission following a stroke event (8). Nearly half of acute stroke patients are readmitted within one year after their stroke episode (9). Approximately 10.4% of stroke patients experience prolonged hospitalization, accounting for 38.9% of total person-hospital days and 47.8% of in-hospital medical expenses. In Taiwan, acute stroke care is primarily provided by medical centers and large regional hospitals, and prolonged hospitalization often leads to bed occupancy in these large-scale hospitals (10).

**Figure 1:**
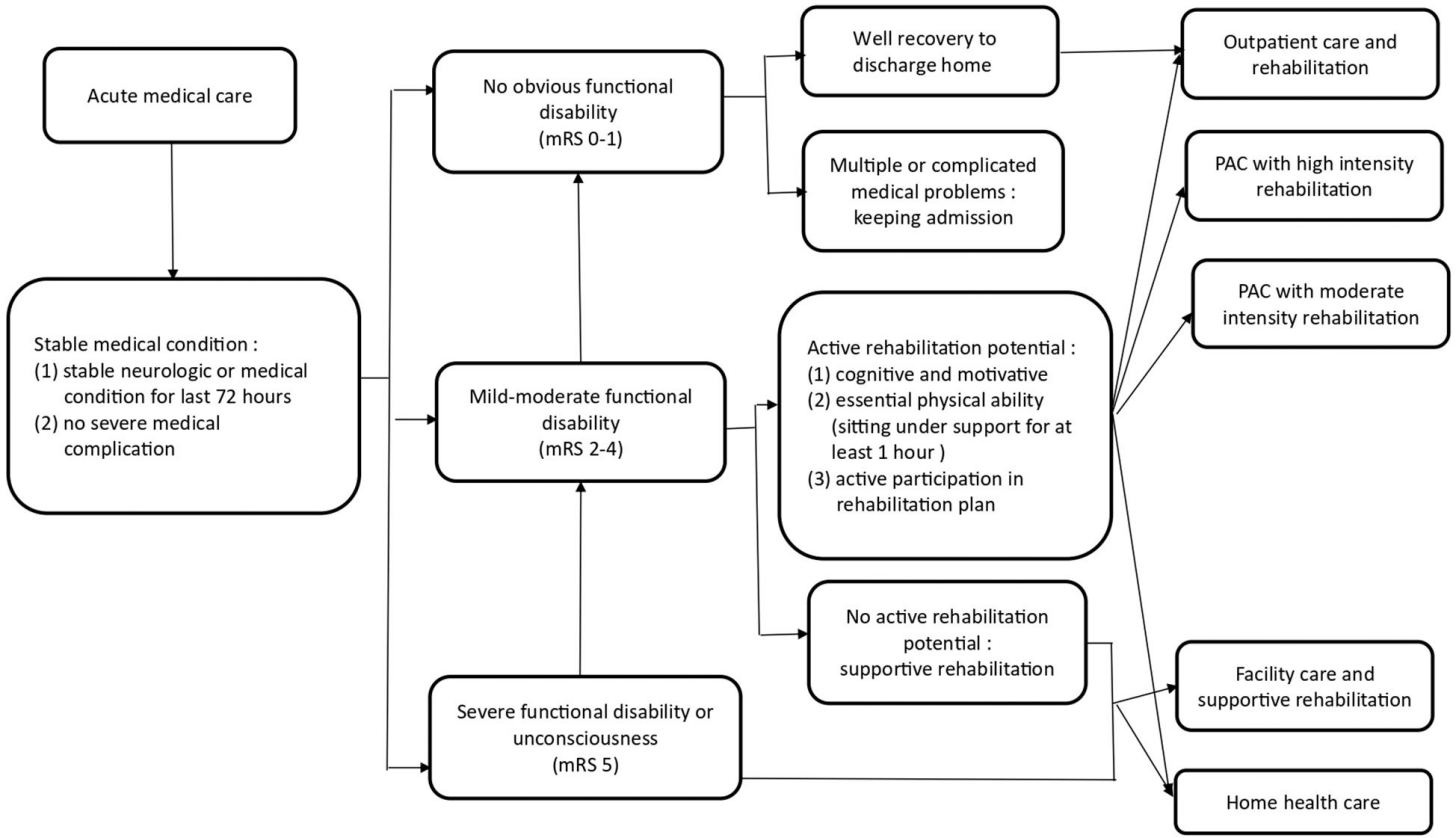
Flowchart of the PAC-CVD program.

**Figure 2:**
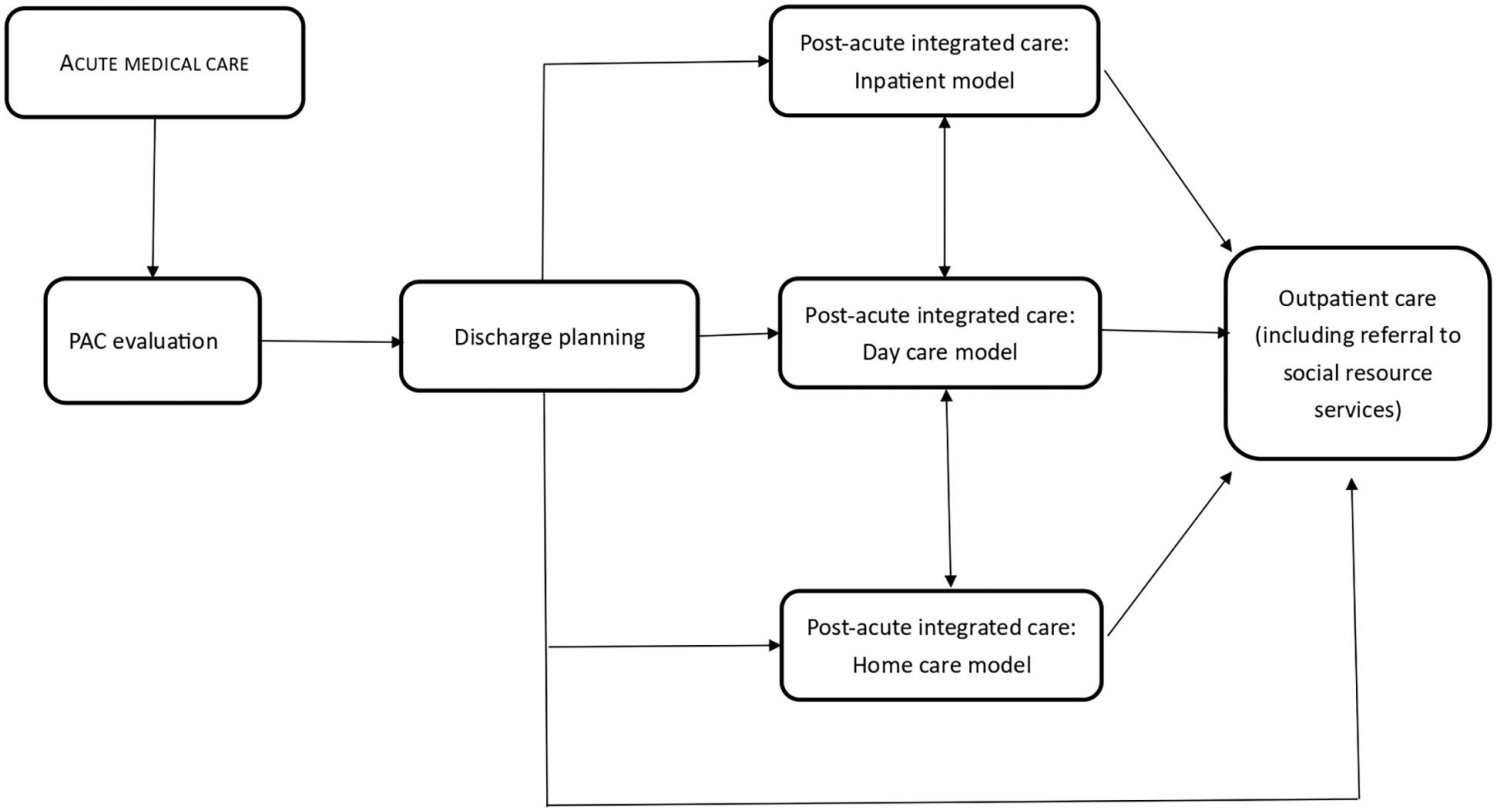
PAC integrated program beginning in 2017.

The National Health Insurance Administration (NHIA) of the Ministry of Health and Welfare of Taiwan implemented the Post-acute Care-Cerebrovascular Diseases (PAC-CVD) pilot program in 2014 and the comprehensive PAC-Integration program in 2017 (Figures 1, 2). The PAC-CVD program was intended to facilitate functional recovery of stroke patients, reduce length of hospitalization and readmission rates, and enable patients to return to their homes and communities sooner. The NHIA recruits community hospitals to provide PAC rehabilitation training programs for acute stroke patients after acute care, incentivizing them by adjusting the payment system from a fee-for-service to a per-diem model and by offering performance payments if the PAC hospitals meet their admission quota. Establishing a vertical integrated healthcare system also facilitates the transfer of patients from large medical centers to community hospitals. Each PAC team comprises one main hospital, typically a medical center or large regional hospital, and various numbers of conducting hospitals, which are medium-scaled regional or small district hospitals within the same geographical area. The transfer of stroke patients within the same hospital (intra-hospital) or to a different conducting hospital (inter-hospital) were the two parts of the referral system. For patients enrolled in the PAC program, if the acute care hospital and the conducting hospital were the same institution, it was defined as an intra-hospital referral. Conversely, if they were different institutions, it was defined as an inter-hospital referral. The PAC hospital must assemble a multidisciplinary rehabilitation team to deliver high-intensity or moderately high-intensity rehabilitation programs that are tailored to the individual conditions of acute stroke patients.

The inclusion criteria for the PAC-CVD program are an acute stroke occurring within the last 30 days, a stable medical condition, Modified Rankin Scale (mRS) scores between 2 and 4, and active rehabilitation potential (Figure 1). The rehabilitation training program is conducted with a frequency of 3–5 sessions per day, with a basic duration of hospital stay in the PAC setting of 3–6 weeks, which can be extended to a maximum of 12 weeks in total, following a thorough review of the patient's progress in functional improvement, as approved by the NHIA.

The functional assessment tools employed in this program comprise a total of 13 evaluation items, namely mRS, Barthel Index (BI), Functional Oral Intake Scale (FOIS), Mini Nutrition Assessment-short form (MNA), Euro QoL-5 Dimensions (EQ-5D) questionnaire, Lawton-Brody IADL Scale (IADL), Berg Balance Scale (BBS), Gait Speed (GS), 6-Minute Walk Test (6MWT), Fugl-Meyer Assessment—Motor & modified Sensation scales (FMA-M & FMA-S), Mini Mental State Assessment (MMSE), Motor Activity Log-Quality of movement scale & Amount of use scale (MAL-Q & MAL-A), and the Concise Chinese Aphasia Test (CCAT). The quality of the PAC program is monitored by the NHIA through various metrics, including functional assessment scales of patients, transfer rate to ICU during PAC admission, 14-day and 30-day readmission rates following the PAC program, and 1-year mortality rate post-PAC. These metrics are essential for assessing the effectiveness and quality of PAC services.

The introduction of the PAC-CVD program can maximize stroke care capabilities of community hospitals, encompassing both regional and district hospitals, and provide inpatient rehabilitation therapy to improve the accessibility of healthcare for stroke patients. The PAC-CVD program was launched in Taiwan in 2017 and, since then, dozens of studies on it have been published. The studies in this review mainly focused on the impact of the PAC program on outcomes of stroke patients, quality of care, influence of referral system, predictive factors for patient outcome, and the cost-effectiveness of the PAC-CVD model. This narrative synthesis is aimed at exploring the overall effectiveness of the PAC-CVD program in Taiwan over the last decade in a review setting.

## Materials and methods

### Articles review process & data sources

**Figure 3:**
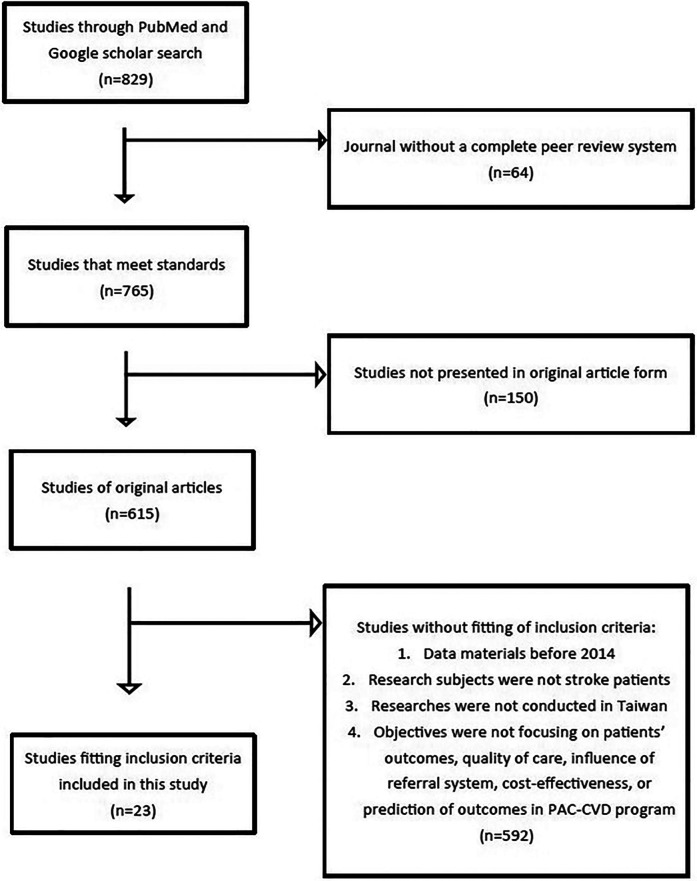
The flow chart of the inclusion and exclusion processes in this study.

We conducted a systemic review of PAC implementation in Taiwan from 2014 to 2023. PubMed and Google Scholar databases were searched for published articles relevant to this study. The combined search terms were “Stroke” AND “Post-acute care” AND “Taiwan”. The search engine incorporated articles with key search terms in titles, abstracts, or texts of articles. The flow chart of inclusion and exclusion processes in this study was presented in Figure 3. The inclusion criteria for articles selection were (1) studies exploring stroke care, (2) studies involving post-acute care, (3) studies conducted in Taiwan, (4) studies focusing on inpatient models of the PAC-CVD program, (5) studies with either a quantitative or qualitative design, and (6) studies published from January 01, 2014 to June 30, 2023. The research articles were independently extracted by multiple observers following the guidance of article objectives focusing on outcomes of patients, quality of care, influence of referral system, cost-effectiveness, or outcome prediction of the PAC-CVD program. This study conforms to all PRISMA guidelines and reports the required information accordingly.

## Results

Through the review process, 829 articles were retrieved initially. After retrieval, 64 articles were excluded because their journals lacked a complete peer review system, and another 149 because they were not original. A further 592 were not included in this study because they did not focus on patients’ outcome, quality of care, the influence of the referral system, cost-effectiveness, or predictive factors for outcomes. Finally, 23 studies were incorporated in our study (Figure 3). Table 1 summarizes authors, years of publication, sample sizes, study designs, and the major findings of recruited studies in this review.

**Table 1:** Sample size, study design, and major findings of studies in this review.

Author	Year	Sample size	Study design	Study objectives (SO)/Major findings (MF)
Lai	2017	168	Retrospective cohort study	SO: To evaluate functional outcomes of PAC programs
				MF: Significant improvement in mRS, BI, IADL, FMA, FOIS, EQ-5D, BBS, and MMSE
Peng	2017	657	Propensity score-matching study	SO: To evaluate the benefits on clinical outcomes and subsequent healthcare utilization
				MF: 1. Significant improvement in all 13 measurement indicators
				2. Significant lower 90-day readmission and ER visit rates in PAC than non-PAC patients
Wang	2017	181	Longitudinal prospective cohort study	SO: To explore how PAC in varying hospitalization paths affects medical care utilization and functional outcomes
				MF: 1. Patients referred from regional hospitals had lower medical care utilization than medical centers
				2. No difference in functional outcomes between regional hospitals and medical centers
Hsu	2019	45	Retrospective cohort study	SO: To explore functional outcomes of PAC programs
				MF: Significant improvement in all 13 measurement indicators
Wang	2019	483	Propensity score-matching study	SO: To assess the impact of the medical referral system and longitudinal effect on functional outcomes
				MF: The intra-referral pattern had better functional outcomes and shorter LOS than inter-referral pattern.
Chien	2020	95	Retrospective cohort study	SO: To investigate functional recovery of the PAC program
				MF: 1. Significant improvement in mRS, BI, IADL, FOIS, EQ-5D, BBS, and MMSE
				2. Significant removal rates for nasogastric tubes and Foley catheters
Tung	2021	193	Retrospective cohort study	SO: To compare the cost-effectiveness and functional recovery between home-based and inpatient-based PAC
			(two-group comparison)	MF: 1. The total rehabilitative cost was cheaper in the home-based model for BI score
				2. The cost-effectiveness was better in the home-based model than inpatient model
Weng	2022	267	Retrospective cohort study	SO: To determine PAC assessment parameters for predicting clinical outcomes
			(Multi-centers cohorts)	MF: 1. Significant improvement in mRS, BI, IADL, BBS, and MMSE
				2. Higher baseline function and greater improvement were related to lower readmission and mortality rates
				3. LOS was related to improvements of ADLs, FOIS, MNA, FMA-motor, and MMSE scores
Chang	2022	334	Muti-centers retrospective cohort study	SO: To compare the effectiveness of PAC with traditional inpatient rehabilitation for stroke patients
			(two-group comparison)	MF: The PAC group showed better improvements in BI, MNA, EQ-5D, IADL, and MMSE than non-PAC group
Tung	2021	197	Retrospective cohort study	SO: To compare the inpatient PAC model with the home-based PAC model in cost-effectiveness and functional recovery
			(two-group comparison)	MF: The inpatient model had better improvement in QoL than the home-based model.
Huang	2019	346	Retrospective cohort study	SO: 3-month follow up of functional recovery of PAC by mRS compared to traditional rehabilitation
			(PAC and case-matched control group)	MF: The PAC group had similar frequency of better functional recovery but less frequency of worse outcomes
Chiu	2021	273	Prospective cohort with propensity score-matched study	SO: To examine longitudinal changes of functional status and predictors
				MF: 1. Higher functional scores in PAC than non-PAC and between-group differences increased over time
				2. Age, hemorrhagic stroke, and poor function scores before rehabilitation were risk factors for poor outcomes
Chiou	2022	41,921	Population-based retrospective cohort study	SO: To explore the probabilities and predictors of 30-day and 1-year potentially preventable readmission
				MF: The ORs of long-term PPR showed a decreasing trend since implementing the PAC program in 2014
Chiu	2021	910	Prospective cohort with propensity score-matched study	SO: To evaluate the impact of PAC programs on cost and functional status
				MF: 1. Total direct medical cost was significantly lower in PAC than non-PAC
				2. Functional recovery was significantly better in PAC than non-PAC, even at 1-yr follow-up
Chen	2022	240	Multi-centers prospective cohort with propensity score-matching study	SO: To measure cost utility and functional outcomes of the PAC program
				MF: 1. The PAC group showed better improvements in BI, EQ-5D, IADL, BBS, and MMSE than non-PAC group
				2. The PAC group had significantly lower direct medical cost and higher gain of QALY than non-PAC group
Chou	2023	263	Retrospective cohort study	SO: To investigate changes in functional performance and NHI cost over 12 PAC hospitalization weeks
				MF: 1. All functional performance measures demonstrated significant improvements over weeks 0–12.
				2. Functional performance had no significant impact on NHI cost at any time point
Hung	2017	178	Prospective cohort study	SO: To determine whether the CARE-C scores could predict the LOS of PAC patients
				MF: 1. Indwelling urinary catheter placement status at admission was a positive predictor of the LOS
				2. Age, core transfer subscale score at admission, and difference in continence subscale score were negative outcome predictors
Tung	2021	193	Retrospective cohort study	SO: To investigate whether the LOS in PAC correlates with functional improvements
				MF: Duration of PAC stay was significantly positively correlated with BI, BBS, gait speed, and FMA-M & FMA-S
Wang	2020	316	Prospective cohort with propensity score-matched study	SO: To compare how PAC affects recovery of walking ability and other functions in different age groups
				MF: 1. The younger group had significantly larger improvements in BBS, IADL, EQ-5D, & 6MWT
				2. No differences in BI, gait speed, LOS, or cost
Peng	2019	1,522	Retrospective cohort study	SO: To evaluate QoL and possible predictors of middle-aged and older stroke patients receiving PAC
				MF: 1. EQ-5D utility: less than 50 Y/O group > 75–84 Y/O group > over 85 group
				2. EQ-5D utility of PAC service duration rose by 0.0733 per one incremental day
Chu	2023	573	Retrospective cohort study	SO: To examine the degree to which characteristic and functional ability could predict patient gait performance
				MF: 1. Patients who regained walking ability earlier had a higher chance of achieving higher levels of walking activity
				2. BBS, age, NIHSS, MNA, and FMA could predict patient's gait speed at discharge
Liao	2021	120	Retrospective cohort study	SO: To investigate the association between admission clinical parameters and community ambulation (6MWT) at discharge
				MF: 1. The BBS on admission was the only significant predictor for community ambulation
				2. The optimal cut-off score of BBS at admission was 29 for community ambulation
Chen	2023	1,988	Population-based retrospective cohort study	SO: To investigate the association between hospital partnership and patient outcomes in PAC
				MF: 1. A dose-response relationship was observed between collaborative strength of hospitals and patient outcomes
				2. Hospital pairs with highly or moderately concentrated referrals had lower readmission and mortality risk

PAC, post-acute care; mRS, modified rankin scale; BI, Barthel index; FOIS, functional oral intake scale; MNA, mini nutrition assessment; EQ-5D, euro Qol-5 dimensions questionnaire; IADL, lawton-brody instrumental activities of daily living scale; BBS, berg balance scale; GS, gait speed; 6MWT, 6-minute walk test; FMA-M & FMA-S, Fugl-Meyer assessment- motor and modified sensation scales; MMSE, mini-mental state examination; LOS, length of stay; PPR, potentially preventable readmission; NHI, national health insurance; NHISS, national institutes of health stroke scale; CARE-C, Chinese version of the continuity assessment record and evaluation; QoL, quality of life.

### Functional recovery

Table 2 demonstrated the functional recovery of stroke patients, comparing performance of evaluation tools before and after the PAC-CVD program. 10 studies investigated the performance of different assessment indicators. Of these 10 studies, eight showed significant improvement in all assessment indicators evaluated and two found non-significant changes in one–two indicators (11–20). PAC rehabilitation training resulted in significant improvements in most assessment tools involved in these studies, including MRS, BI, EQ-5D, FOIS, MNA, BBS, GS, 6MWT, FMA, and MAL; however, some studies showed no statistical significance with some evaluation tools. Wang's investigation in 2019 illuminated a difference of 0.43 points among stroke patients undergoing intra-hospital transfer patterns, showing a significant improvement in IADL (15). However, in the case of inter-hospital transfer patients, there was a difference of 0.21 points, which did not reach statistical significance. Notably, the magnitude of amelioration in IADL observed in both intra- and inter-hospital transferring cohorts was comparatively lower when juxtaposed with the findings of other studies. Eight studies examined MMSE for cognitive function and demonstrated significant improvement, ranging from 1.47 to 5.0, although the patients in the inter-hospitals transfer pattern group (1.14) of Wang's study showed no significant improvement (15). Lai's study in 2017 demonstrated an improvement in the CCAT score by 0.51 but also lacked statistical significance (11).

**Table 2:** Functional recovery in PAC-CVD programs.

Authors	Lai, etc.	Peng, etc.	Wang, etc.	Hsu, etc.	Wang, etc.		Chien, etc.	Tung, etc.	Weng, etc.	Chang, etc.	Chen, etc.
Publication year	2017	2017	2017	2019	2019		2020	2021	2022	2022	2023
Case No.(*n*=)	168	657	181	45	483 (intra*)	483 (inter)	95	197	267	122	73
Mean LOS	43.57	15.0	27.6	63	35.75	36.5	58.15	35.01	–	48.73	30.47
ΔmRS	−0.45 (V)	–	−0.873 (V)	−0.6 (V)	–	–	−0.84 (V)	−0.554 (V)	−1.0 (V)	–	−0.86 (V)
ΔBI	26.16 (V)	24.1 (V)	24.475 (V)	23 (V)	9.59(V)	7.61 (V)	34.21 (V)	25.839 (V)	30.0 (V)	25.78 (V)	28.42 (V)
ΔIADL	1.5 (V)	1.0 (V)	1.464 (V)	1.7 (V)	0.43 (V)	0.21 (X)	1.35 (V)	1.352 (V)	1.0 (V)	1.20 (V)	1.58 (V)
ΔEQ-5D	−0.314 (V)	–	−1.961 (V)	−0.58 (V)	−0.86 (V)	−0.38 (V)	–	−0.3844 (V)	−0.4 (V)	−0.23 (V)	−1.95 (V)
ΔFOIS	0.85 (V)	0.7 (V)	0.420 (V)	1.5 (V)	0.03 (V)	0.3 (V)	1.32 (V)	0.495 (V)	1.0 (V)	0.72 (V)	–
ΔMNA	1.98 (V)	2.0 (V)	–	–	–	–	–	1.832 (V)	2.7 (V)	1.11 (V)	–
ΔBBS	17.50 (V)	15.3 (V)	16.7 (V)	14.1 (V)	11.02 (V)	7.49 (V)	17.31 (V)	15.534 (V)	20.5 (V)	–	–
ΔGS	–	1.85 (V)	–	0.23 (V)	–	–	–	4.726 (V)	–	–	–
Δ6MWT	–	41.8 (V)	–	54 (V)	–	–	–	91.443 (V)	–	–	–
ΔMMSE	3.11 (V)	2.9 (V)	–	6 (V)	1.47 (V)	1.14 (X)	3.57 (V)	3.156 (V)	5.0 (V)	3.37 (V)	–
ΔFMA-S	–	5.7 (V)	–	9 (V)	–	–	–	9.358 (V)	5.0 (V)	–	–
ΔFMA-M	–	9.8 (V)	–	10 (V)	–	–	–	11.757 (V)	12.0 (V)	–	–
ΔMAL-A	–	0.7 (V)	–	1.3 (V)	–	–	–	0.679 (V)	–	–	–
ΔMAL-Q	–	0.8 (V)	–	0.9 (V)	–	–	–	0.278 (V)	–	–	–
ΔCCAT	0.51 (X)	0.6 (V)	–	1.3 (V)	–	–	0.83 (V)	0.680 (V)	0.5 (V)	–	–

mRS, modified Rankin Scale; BI, barthel index; FOIS, functional oral intake scale; MNA, mini nutrition assessment; EQ-5D, euro Qol-5 dimensions questionnaire; IADL, lawton-brody instrumental activities of daily living scale; BBS, berg balance scale; GS, gait speed; 6MWT, 6-minute walk test; FMA-M & FMA-S, Fugl-Meyer assessment-motor and modified sensation scales; MMSE, mini-mental state examination; MAL-Q& MAL-M, motor activity log—quality of movement scale & amount of use scale; CCAT, concise Chinese aphasia test.

* Intra: intra-hospital transferring pattern; inter: inter-hospital transferring pattern.

(V): statistically significant; (X): not statistically significant.

Δ: difference before and after PAC-CVD rehabilitation program.

Table 3 demonstrated the comparison of functional recovery between PAC and non-PAC stroke patients and showed that all functional outcomes (BI, FOIS, MNA, EQ-5D, IADL, BBS, and MMSE) measured before discharge demonstrated significant improvements in both PAC and non-PAC groups compared with the values upon admission. PAC patients demonstrated more significant improvements in all assessed outcome variables than non-PAC patients, except FOIS in Chang's study (19).

**Table 3:** Comparison of functional improvement between PAC and non-PAC groups.

Authors	Chang, etc.	Chiu, etc.	Chiu, etc.*
Year of publication	2022	2021	2021
*n* = (PAC/non-PAC)	122/212	273/273	455/455
^†^ΔBI (PAC/non-PAC)	25.78/15.87 (V)	7.42/3.41 (V)	19.3/17.3 (V)
ΔIADL (PAC/non-PAC)	1.20/0.11 (V)	1.27/1.19 (V)	1.1/0.2 (V)
ΔEQ-5D (PAC/non-PAC)	−0.23/−0.17 (V)	−2.13/−0.80 (V)	−0.8/0 (V)
ΔFOIS (PAC/non-PAC)	0.72/0.58 (X)	1.01/0.55 (V)	0.3/0 (V)
ΔMNA (PAC/non-PAC)	1.11/0.67 (V)	–	
ΔBBS (PAC/non-PAC)	–	8.94/5.96 (V)	9.3/6.5 (V)
ΔMMSE (PAC/non-PAC)	3.37/2.13 (V)	1.74/1.36 (V)	2.2/1.0 (V)

BI, Barthel index; IADL, lawton-brody instrumental activities of daily living scale; EQ-5D, euro Qol-5 dimensions questionnaire; FOIS, functional oral intake scale; MNA, mini nutrition assessment; BBS, berg balance scale; MMSE, mini-mental state examination.

* Significance identified by comparison of differences of effect sizes between PAC and non-PAC groups after rehabilitation.

† ΔDifference of Functional improvement between PAC and non-PAC groups.

Huang's study utilized a case-control method to compare mRS scores of 173 cases in PAC and non-PAC groups as a functional recovery index (21). By assessing functional recovery using the three-month mRS score compared with initial status, it was shown that the PAC group exhibited a higher percentage of better outcomes than the non-PAC group (40.4% vs. 33.9%). A smaller decline in functionality was reported in the PAC group compared with patients in the non-PAC group (1.8% vs. 5.8%). The PAC group demonstrated a higher rate of favorable functional recovery and a lower rate of unfavorable recovery compared to the control group, suggesting a potential benefit or protective effect of the PAC intervention.

Chiu's study investigated longitudinal changes of functional outcomes for the 12-week PAC rehabilitation program and at 1-year follow-up (22). This study showed that the PAC group had better functional improvement, and differences in all functional outcome measures (BI, IADL, FOIS, EQ-5D, MMSE, & BBS) between the two groups increased significantly over time from baseline to 1-year follow-up.

### Quality of care

Table 4 presents the quality of stroke care in PAC programs, as reported in various studies over the past decade. The readmission rates for stroke patients within 14 days after transferring to PAC hospitals were reported as 3.8% in Hsu's study (14) and 5.6% in Lai's study (11), which were notably lower than the previous finding of 17.6% found in stroke patients discharged from acute stroke care hospitals without the PAC program.

**Table 4:** Quality of stroke care in PAC and non-PAC groups.

Outcomes of care quality	Authors	PAC group	Non-PAC group	Significance^a^
14-day readmission rate	Lai, etc.	5.6%	–	
	Hsu, etc.	3.8%	–	
	Weng, etc.	4.9%	–	
30-day readmission rate	Chiou's, etc.	15.48%		
90-day readmission rate	Peng, etc.	11.1%	21.0%	V
90-day stroke-related readmission rate	Peng, etc.	2.1%	8.8%	V
1-year readmission rate	Chiou's, etc.	47.25%	–	
90-day mortality rate	Peng, etc.	1.4%	2.0%	X
1-year mortality rate	Weng, etc.	3.7%		
90-day ER-visit rate	Peng, etc.	13.5%	24.0%	V
Home-returning rate	Lai, etc.	76.8%	–	
	Hsu, etc.	73.0%	–	

V, significant; X, not significant.

a PAC group comparing to non-PAC group.

Peng's study (2017), employing a propensity-scored matched case-control method, showed that patients in the PAC group had significantly lower 90-day readmission rates (11.1% vs. 21.0%, adjusted odd's ratio, aOR: 0.47), stroke-related admissions (2.1% vs. 8.8%, aOR: 0.22), and ER visits rates (13.5% vs. 24.0%, aOR: 0.49) compared with patients in the control group. However, the differences in the 90-day mortality rates between both groups were insignificant (1.4% vs. 2.0%) (12).

Chiou's study investigated potential predictors of 30-day and 1-year potentially preventable readmission (PPR) among 41,921 first-stroke patients using claims data from the NHIA between 2010 and 2018 (23). In this study, 30-day and 1-year readmission rates were 15.48% and 47.25%, respectively, and PPR & non-PPR were 9.84% and 5.65% within 30 days and 30.65% and 16.60% within 1-year, respectively. A descending trend in ORs of long-term PPR compared to non-readmission was noted since the implementation of the PAC-CVD program in 2014. A dramatic decline in 2018 was attributed to the expansion of the long-term care plan to a newer version—Long Term Care (LTC) 2.0—in 2017 by Taiwan's government, which greatly expanded the resources for and availability of long-term care.

Lai's study (11) and Hsu's study (14) showed, respectively, that 76.8% and 73.0% of stroke patients in the PAC program returned to their homes and communities after discharge from PAC hospitals. Data publicly released by Taiwan's NHIA at a PAC forum revealed that 87.5% of stroke patients participating in the PAC program achieved improved functionality and 83.7% returned to their homes and communities after discharge.

### Efficiency and cost-effectiveness

The PAC program has the potential to reduce the medical expenditure of acute stroke patients and be more cost-effective than traditional inpatient rehabilitation models. The total direct medical cost of a PAC patient with a per-diem based payment was lower than that of a non-PAC patient with a fee-for-service based payment. In Chiu's study (2021), the total direct medical cost of each patient in a PAC group was 4,790.3 $USD lower than that in a non-PAC group ($4,139.5USD vs. $8,929.8USD), and total direct medical cost after discharge of each PAC patient was 58.8 $USD lower than non-PAC patient ($1,187.2USD vs. $1,246.0USD) (24). Chen et al. analyzed Cost-Utility of the PAC-CVD program, revealing a significantly lower mean direct medical cost of PAC groups ($3,480USD vs. $3,785USD, *P* < 0.001) and a higher average gain of quality-adjusted life year (0.1993 vs. 0.1233, *P* < 0.001) than non-PAC groups (25). The duration of PAC hospital stay would influence medical expenditure, too. Chou's study investigated the medical cost of different time points of PAC hospitalization and found NHI costs varied from $1,695.53 USD to $2,060.84 USD for every 3 weeks of hospitalization time points (26). Changes in NHI costs varied depending on whether hospitalization was extended, so that functional performance had no significant impact on NHI costs at any time point.

The cost-effectiveness of PAC programs varies from model to model. The home-based model was more cost-effective than the inpatients model, whereas the functional recovery improvements remained similar between the two. Tung reported that medical expenditure (total rehabilitation cost) was less in the home-based PAC model than in the inpatient PAC model (17). Cost-effectiveness for BI, IADL, MNA, and EQ-5D was better in the home-based model than in the inpatient model. For example, the cost-effectiveness for per point of BI increase was about $152.47 USD in the inpatient group and $48.18 USD in the home-based group. The rehabilitation hours for each point increase of BI score was less in the home-based model than in the inpatient model.

### Outcome prediction

Several studies have investigated potential predictors for functional recovery and outcomes. Weng et al. investigated the impact of functional assessment tools on outcome prediction of stroke patients receiving PAC (18). Of the 13 functional assessment tools, 11 were utilized in this study, excluding gait speed and 6MWT. It was found that stroke patients with a higher baseline function and greater improvement of physical and cognitive function after training in PAC wards had lower 14-day readmission and 1-year mortality rates. Reduced mortality and readmission rates were associated with improved MMSE & functional improvements in at least five of the 11 assessment tools. LOS was related to improved scores in BI, FOIS, MNA, FMA-motor, and MMSE scores. Prolonged LOS was associated with improved FOIS, MNA, and functional improvements in at least seven of the 11 assessment tools. Hung's study utilized the Chinese version of the Continuity Assessment Record and Evaluation to evaluate functional status of patients across different acute and PAC settings and found that indwelling urinary catheter placement status at admission was a significant positive predictor for LOS. Age, core transfer subscale score of Functional Independent Measure at admission, and difference in continence subscale score were negative predictors for LOS (27). In contrast, Tung et al. (2021) revealed that the duration of PAC hospitalization was significantly positively correlated with functional outcomes, including BI, BBS, 5MWS, and FMA-Motor and Sensation (28). Age is suggested to be a prognostic indicator for the outcomes of stroke patients. Wang's study investigated functional recovery in different age groups after a PAC program, showing significant improvements in BI, EQ-5D, BBS, 5MWS, and 6MWT in both aged (≧65 Y/O) and non-aged (<65 Y/O) stroke patients (29). The non-aged group had significantly better improvements in BBS, IADL, EQ-5D, and 6MWT than the aged group, and no significant differences in BI, 5MWS, or LOS in either group were shown. Peng' study evaluated health-related QoL of middle-aged and older stroke patients receiving PAC and found the EQ-5D utilities in 75–85 and over 85 age groups were 0.091 and 0.159 lower respectively than those younger than 50 (30). In addition to age, patients with higher BI or no previous stroke history had better utilities gains than those with lower BI or previous stroke history. The EQ-5D utility in PAC duration increased by 0.0733 for every incremental day. In addition, stroke type was another factor influencing outcomes. Chiu's study found age, hemorrhagic stroke, and poor functional status before rehabilitation were risk factors of poor functional recovery for stroke patients in a PAC program (22).

Chu's study found stroke patients in PAC programs who regained their walking ability earlier (completing a 5MWT) had a higher chance of achieving higher levels of walking activity. Age, BBS, leg motor drift score of NIHSS, FMA, and MNA could predict gait speed of stroke patients at discharge from PAC wards (31). Moreover, community ambulation (walking distance ≧205 meters in 6MWT) is an important goal for stroke patients. Liao's study revealed that BBS score at admission was the only significant predictor for community ambulation in stroke patients on a PAC program, and it was not affected by age, sex, stroke type, LOS, NG/foley tube use, 5MWT, MMSE, or MNA (32). The cut-off point of BBS score at admission for community ambulation at discharge is 29 and the area under ROC curve for BBS score when discriminating between household and community ambulation at discharge was 0.74.

### Impact of referral system

Intra-hospital referral of stroke patients participating in PAC programs is associated with more favorable functional recovery and quality of care than those with inter-hospital referral. Wang's study investigated the impact of different referral systems on functional recovery of stroke patients receiving PAC rehabilitation training and found that functional outcomes (BI, IADL, FOIS, MMSE, BBS, and EQ-5D) of intra-hospital referral patients had significantly better improvement than those of inter-hospital referral patients (15). The MMSE and IADL even showed no significant improvement in inter-hospital transferal patients. The average duration of PAC hospital stay for intra-hospital referral patients was significantly shorter than for patients in the inter-hospital referral system (31.52 days vs. 37.1 days, *p* < 0.001). The mean LOS of stroke patients in an acute care unit before referral to PAC settings was significantly shorter in an intra-hospital referral group compared with an inter-hospital referral groups (13.01 days vs. 24.45 days, respectively), too (15). This suggests that the referral system may have an impact on overall outcomes of stroke patients participating in PAC-CVD programs. Wang also analyzed functional outcomes and mean duration of stroke patients in PAC hospitals with different levels of acute care hospitals (13). The functional outcomes (BI, IADL, FOIS, EQ-5D, and BBS) of stroke patients at different levels of acute care hospitals showed improvements but without a significant difference, and the mean LOS in PAC hospitals and acute care units before referral were both lower in regional hospitals compared to medical centers (13).

The partnership between acute care and PAC hospitals influences patient outcomes. Chen (2023) revealed that stronger collaboration between acute care and PAC hospitals substantially improved post-discharge patient outcomes (33). A dose-response relationship was observed between collaboration levels of hospitals and patient outcomes. Moreover, referral concentration also had an impact on patient outcomes. Stroke patients in hospitals paired with highly or moderately concentrated referrals and strong relationships had lower readmission and mortality rates. A greater number of shared patients and a more concentrated referral linkage between acute and PAC hospitals reduced potential adverse outcomes of stroke patients within a PAC program.

## Discussion

### Functional recovery

The main purpose of the PAC-CVD program is to enhance functional recovery of acute stroke patients and facilitate their return to their homes and communities. Studies conducted in Taiwan and published in the past 10 years have consistently demonstrated variable degrees of functional improvement across various evaluation tools, as depicted in Table 2. Most studies showed significant improvements in functional recovery and demonstrated that intensive rehabilitation training in PAC-CVD programs resulted in various degrees of improvement in all dimensions of function recovery, including general severity of the disease, ability of daily activity, QoL, swallowing function, nutritious status, balance, cardiopulmonary fitness, walking ability, motor function and sensation of upper extremities, cognition, and speech ability.

Peng's study highlighted that among various functional outcomes, balance (as measured by BBS) exhibited the most significant improvement. An improvement in balance is crucial as it may contribute to a reduction in the risk of subsequent falls and related injuries (12). The overall pattern of speech function showed that the majority of studies revealed positive changes as measured by CCAT following PAC interventions, emphasizing potential effectiveness for speech training programs. The lack of statistical significance for CCAT in Lai's study and lower magnitudes of FOIS in Wang's study suggested some variabilities in observed outcomes across studies with different designs (11, 15).

Comparative studies between PAC and traditional inpatient rehabilitation programs could contribute to a better understanding of the strengths and limitations of each approach, potentially informing decisions regarding the optimal rehabilitation strategy for acute stroke patients. In addition to case series studies comparing changes between baseline and post-PAC, several studies conducted comparisons of patients receiving PAC and traditional rehabilitation programs, providing valuable insights into the relative effectiveness of PAC. Table 3 demonstrated better performance of functional recovery in stroke patients receiving PAC rehabilitative programs than those without PAC, including in BI, IADL, EQ-5D, FOIS, MNA, BBS, and MMSE. The PAC-CVD program provided a higher intensity rehabilitation training program with 3–5 sessions of physical, occupational, or speech therapy per day than traditional inpatient rehabilitation programs, leading to better functional recovery. These studies could be particularly useful for healthcare professionals and policymakers seeking to optimize strategies for the delivery of rehabilitation services and for individuals recovering from strokes to improve functional outcomes.

### Quality of care

The PAC-CVD program is designed to enhance the quality of healthcare for stroke survivors by reducing readmission and mortality rates and restoring independence and autonomy in their daily lives. The NHIA evaluates the quality of PAC-CVD using various indicators, assessing the effectiveness and efficiency of the program, ensuring it meets the intended objectives and delivers high-quality care to stroke survivors.

Stroke patients participating in a PAC-CVD program exhibited lower readmission rates within 14 days of transferring to PAC hospitals and shorter stays in PAC wards than patients in non-PAC programs (12). The findings suggest that practicing PAC in the care pathway for stroke patients may contribute to lower readmission rates within both the early post-transfer and entire PAC admission period. Table 4 showed the same phenomenon: the implementation of PAC programs for stroke patients was associated with lower rates of readmission and ER visits post-discharge in comparison to those without PAC. After discharge from acute hospitals, stroke patients can exhibit persistent instability in their medical and physical conditions due to sequelae of functional impairment, which might lead to medical condition decline or complications, such as pneumonia, urinary tract infections, or falls. The PAC model composes quality medical care and an intensive rehabilitative training program to prevent complications and reduce impairment and disability, resulting in reduced readmission and mortality rates and ER use after discharge.

Chiou's study reported a decline in long-term PPR from 2014 (after the implementation of PAC) and a dramatic decline from 2018 (after the expansion of the long-term care resources and availability of LTC-2.0 implementation in 2017) (23). The combination of intensive rehabilitative training, an integrated discharge plan, and multi-resource allocation of long-term care is beneficial in functional recovery, reduces the risk of complications and the need for individualized care plans and coordination of post-discharge care, and improves overall well-being. Taken together, PAC not only contributes to immediate post-stroke care but also has long-term benefits in terms of reducing readmissions and improving QoL for stroke survivors. The above studies suggest that PAC rehabilitative training programs could reduce subsequent readmission and mortality, decrease healthcare utilization after discharge, and, in turn, improve the quality of healthcare for stroke patients. An intensive PAC rehabilitation model may be taken into account to improve the standard of care of stroke patients to maximize functional recovery and quality of life.

### Efficiency and cost-effectiveness

The NHIA changed the payment protocol for the PAC-CVD program from a fee-for-service model to a per-diem model. The NHIA subsidizes the cost of PAC by $110–115 USD per weekday and $40–43 USD per weekend or holiday for each patient in an inpatient rehabilitative PAC model. An increasing number of community hospitals are being recruited to the PAC-CVD program to provide rehabilitative care for stroke patients after acute care in large-scale hospitals. It is suggested that the PAC-CVD program reduces the medical expenditure of acute stroke patients and is more cost-effective than the traditional inpatient rehabilitation model (17, 24–26). Moreover, the PAC program contributes to the expansion of stroke care capacity and release of pressure on occupancy of acute beds in large-scale hospitals, leading to improved patient flow and more timely access to acute care services.

In conclusion, the PAC-CVD program's adoption of a per-diem-based payment system not only improves patient outcomes but also has economic advantages by reducing the total and mean direct medical cost for acute stroke patients, as well as affecting post-discharge expenditure. The patients within PAC-CVD programs also had better performance in cost-utility than non-PAC patients (25). These findings suggest a move toward a more cost-effective and sustainable model of healthcare delivery in Taiwan. However, each PAC hospital must assemble a multidisciplinary rehabilitation team consisting of various professionals, including physiatrists and/or physicians, physical therapists, occupational therapists, speech therapists, rehabilitation nurses, pharmacists, dietitians, and social workers, all of whom are instrumental in delivering high-intensity or moderately high-intensity rehabilitation programs that are tailored to the individual conditions of acute stroke patients. The rehabilitative training program is conducted on a frequency of 3–5 sessions per day, with each session encompassing either physical therapy, occupational therapy, or speech and swallowing therapy. The medical professionals should perform a complete evaluation with the 13 assessment tools at admission, every three weeks, and at discharge. The labor-intensive rehabilitation programs and repeated evaluations are time consuming and increase the workload of clinical staff, which makes the costs disproportionate to the high-quality and labor-intensive services. The successful establishment and maintenance of effective PAC programs are largely attributed to the efforts and selfless dedication of frontline healthcare professionals. Policymakers should consider more resource allocation to the PAC program.

### Outcome prediction

Hence, the PAC rehabilitation program can improve functional recovery of and quality of healthcare for stroke patients. Understanding prognostic or predictive factors that influence the outcomes of stroke patients in the PAC program is an important issue for optimizing the effectiveness of rehabilitation strategies and tailoring interventions to meet individual needs. Several studies have delved into these factors to identify predictors of functional recovery and outcomes. The findings of these investigations should be considered by healthcare professionals for their value in treatment planning and development of targeted rehabilitation interventions.

The PAC-CVD program assesses functional recovery and quality of life using 13 tools. The level of baseline condition is associated with degrees of functional outcome after rehabilitative training, as previous studies showed. A favorable improvement in functional recovery, especially physical and cognitive function, after PAC care is associated with reduced subsequent readmission and mortality rates because it decreases falls and medical complications, including pneumonia, hip fractures, and pressure sores. For instance, better swallowing and cognitive function would lead to a safer swallowing process (34), higher nutritious status, and better physical activity, consequently contributing to lower aspiration pneumonia and readmission. Prolonged LOS was correlated with functional improvement; in contrast, duration of PAC hospitalization had a significantly positive impact on functional outcomes. In other words, stroke patients with longer PAC stays in rehabilitative facilities had better ADL, balance, gait speed, and motor and sensory function of the upper extremities (28). Although shorter hospital stays are commonly considered a positive indicator for quality of healthcare, longer rehabilitation duration in PAC settings may lead to better functional improvements and quality of life for stroke patients. It indicates a higher probability for returning home and lower readmission and mortality rates, too.

Age is a significant factor that influences the outcome of stroke patients and quality of care. Different age groups may exhibit variations in their response to rehabilitation interventions and the degree of functional improvement achieved. Younger stroke patients in PAC programs had better functional recovery than aged patients, including balance, IADL, QoL, and cardiopulmonary function (29). The reason for the poor outcome in older patients may be due to poor physical condition for recovery or compensation of brain damage and insufficient socioeconomic support (35). Decreased neuroplasticity in older patients may lead to a decline in the ability to learn new skills (36), and coexisting chronic diseases in these patients may lead to weaker physical functions, too. These factors will affect the speed and extent of recovery. Stroke type has also been shown to have an influence on patient outcome, as previous studies have shown. Patients with ischemic-type stroke had better improvement than patients with hemorrhagic type. Walking ability is important for the mobility of patients, quality of life at home, and social activity or working ability in their community. Besides age, balance, lower extremities’ strength, nutritious status, and upper extremity function were also predictors of walking performance after stroke. Several factors would have an impact on regaining walking ability, although balance function was the only factor with predictive value for community ambulation. Based on the study findings, the rehabilitation team could set the appropriate intervention plans and discharge goals earlier (37). Similar findings were shown in a previous study (38).

### Impact of referral system

The type of referral system appeared to contribute to the quality of care and functional outcomes for stroke patients. Stroke patients in PAC programs with intra-hospital referral had better functional recovery and quality of care, including LOS, than those with inter-hospital referral (13, 15). The intra-hospital referral system could lead to more timely, efficient care of stroke patients with minimal gap in care. The physician and medical professionals have access to detailed information on patients within the same care system. This would have a positive impact on functional recovery and quality of care, as interrupted care and inadequate information about stroke patients may be present in the inter-hospital referral system. However, since medical centers are not authorized to directly provide PAC rehabilitative services, they can only transfer PAC patients to regional or district hospitals. Therefore, PAC cases from medical centers are necessarily inter-hospital referrals. Nevertheless, because medical centers possess higher acute care and treatment capabilities, the patients they admit may tend to have higher disease severity than those in regional or district hospitals, which consequently results in relatively poorer functional recovery in the subsequent rehabilitation phase. As previously mentioned, baseline conditions can significantly influence the functional outcomes, so patients referred from medical centers tend to have poorer functional outcomes. Therefore, poorer functional recovery of inter-referral system patients is not solely due to the referral system but also to the poorer baseline condition of the patient.

Since referral efficiency would have an impact on functional outcome and quality of care of stroke patients in the PAC-CVD program, the partnership between the main responsible and conducting hospitals would also influence patient outcomes. Stronger collaboration and more referral volume and concentration would have a positive impact on post-discharge outcome, including readmission and mortality rates (33). Better collaborative care of stroke patients between acute care units and PAC settings resulted in better outcomes of patients. These findings emphasize the importance of collaboration and coordination between acute care and PAC hospitals in optimizing patient outcomes. Strengthening the relationships and improving the concentration of referrals between these healthcare units may contribute to better overall care and reduced adverse outcomes for stroke patients participating in PAC-CVD programs.

## Conclusion

The NHIA of Taiwan implemented the PAC-CVD program in 2014 by changing the payment system to a per-diem mode and establishing a vertically integrated healthcare system. It incentivized the participation of a broader spectrum of healthcare providers, particularly medium-small-scale regional and district hospitals, and expanded the capacity of stroke care, contributing to a more distributed and accessible network. The PAC-CVD program not only enhanced functional recovery and QoL of acute stroke patients but also improved the quality of healthcare. It offered a more efficient and effective care model for acute stroke patients by reducing medical expenditures. Closer relationship between acute care and PAC hospitals revealed better performance of patient outcomes and quality of care. However, the labor-intensive rehabilitation programs and repeated evaluations are time-consuming and increased the workload of clinical staff, which made the costs disproportionate to the high-quality and labor-intensive services. This study has certain limitations and potential biases originating from study designs or selection biases. Despite this, the successful implementation of PAC-CVD indicates the possibility of a standard rehabilitative care model for acute stroke patients, with expansion to other diseases or conditions after adjustments to the payment structure and heavy workload. It is hoped that this will contribute to the planning of similar policy programs in the future.

## Data Availability

The original contributions presented in the study are included in the article/Supplementary Material; further inquiries can be directed to the corresponding author.
